# Senescence and tumor suppression

**DOI:** 10.12688/f1000research.11671.1

**Published:** 2017-12-11

**Authors:** Philip Hinds, Jodie Pietruska

**Affiliations:** 1 Department of Developmental, Molecular, and Chemical Biology, Tufts University, Boston, MA 02111, USA

**Keywords:** Cellular senescence, tumor suppression mechanism

## Abstract

Cellular senescence has emerged as a potent tumor suppression mechanism that restrains proliferation of cells at risk for malignant transformation. Although senescent cells have permanently exited the cell cycle, their presence can have detrimental effects on the surrounding tissue, largely due to the development of the senescence-associated secretory phenotype (SASP). Here, we review the tumor-suppressive and tumor-promoting consequences of the senescence response, focusing on the SASP as a key mediator of this dichotomy. Accumulating evidence suggests that the persistence of senescent cells can exacerbate the development of a pro-inflammatory, immunosuppressive microenvironment that can favor tumorigenesis. Given that senescence of tumor and stromal cells is a frequent outcome of anti-cancer therapy, approaches that harness the growth inhibitory effects of senescence while limiting its detrimental effects are likely to have great clinical potential.

## Introduction

Limiting the unrestrained proliferation of tumor cells is a primary goal of anti-cancer therapies. Decades of study on cell cycle control and cell cycle arrest have yielded great insight into normal checks on proliferation as well as their dysregulation during tumorigenesis. Cellular senescence has emerged as a multi-dimensional mechanism of proliferative arrest that has pleiotropic effects on a variety of physiological and pathological processes, including but not limited to tumorigenesis. It is now appreciated that senescent cells are beneficial in tissue remodeling during embryogenesis (reviewed in 1) and wound healing ( 2, reviewed in 3) and are capable of conferring senescent phenotypes on neighboring cells in a process termed bystander senescence ^4^. Accumulation of non-proliferative cells over time also has implications for tissue homeostasis, and cellular senescence is now understood to be an important driver of age-related pathologies (reviewed in 5).

The process of cellular senescence has long been recognized as an intrinsic mechanism that limits the proliferative life span of normal cells ^6^. Senescence is a state of permanent cell cycle arrest in which cells remain viable and metabolically active but non-proliferative, even under mitogenic stimulation. Intrinsic, or replicative, senescence is a characteristic of all somatic cells and has been observed in many vertebrate species, including rodents, non-human primates, and humans ^5, 7, 8^. Senescence was first described in human diploid cells as a response to prolonged culture ^6^ and later during growth under high-oxygen conditions ^9^, which initially raised the question of whether senescence was exclusively an *in vitro* phenomenon. To date, copious demonstrations of senescence *in vivo* in both normal and diseased tissue have made clear that this process is biologically relevant. It is now understood that while senescence induction is a potent barrier to tumorigenesis *in vivo*, the presence and persistence of senescent cells can have deleterious effects that contribute to aging and age-related pathologies, particularly cancer ^5^. This dual nature of the senescence response, often referred to as a ‘double-edged sword’, demands careful analysis of risks versus benefits of senescence as a goal of anti-cancer therapies.

## Senescence stimuli and effector pathways

The senescence program can be activated in normal, pre-neoplastic, and malignant cells in response to a wide variety of stimuli. In proliferating somatic cells that lack telomerase expression, replicative senescence occurs as a result of progressive telomere attrition with each subsequent cell division. Critically short uncapped telomeres are recognized as DNA double-strand breaks, activating a classic DNA damage response (DDR) ^10– 12^. Senescence can also be induced independent of telomere dysfunction in response to potentially oncogenic stresses. Genotoxic stress as a result of reactive oxygen species (ROS) generation or exposure to radiation or DNA-damaging agents can also induce the senescence program through the DDR or via p38 MAPK/PRAK signaling ^13, 14^, thereby halting the proliferation of cells harboring mutations or genomic instability. While senescent cells are known to be metabolically active, only recently have alterations in cellular metabolism been causally linked to the establishment of the senescent state. Accumulating evidence indicates that metabolic reprogramming occurs in cells undergoing senescence in response to shortened telomeres (replicative senescence) or activated oncogenes (reviewed in 15). Oncogene-induced senescence (OIS) occurs in response to aberrant hyperproliferative signaling downstream of activated oncogenes (for example, *RAS*, *BRAF*, and *MYC*) ^16– 19^. OIS is a potent tumor suppression mechanism that prevents malignant transformation of pre-neoplastic cells, both in genetic mouse models and in human cancers. Perhaps the most well-studied example of OIS is the formation of benign cutaneous melanocytic nevi (moles), which are composed of senescent melanocytes harboring the oncogenic BRAF ^V600E/K^ mutation ^19^. Malignant transformation of melanocytes requires additional mutations that either prevent or bypass OIS, further underscoring the importance of senescence as a potent tumor-suppressive barrier.

Disruption in higher-order chromatin structure or chromosome ploidy is capable of inducing senescence, including whole chromosome instability (W-CIN) and histone deacetylase inhibition ^20– 22^. Mitochondrial dysfunction was recently identified as a novel stimulus of senescence both *in vitro* and *in vivo*. In human IMR-90 fibroblasts, loss of mitochondrial sirtuins (SIRT3 and SIRT5) triggers mitochondrial dysfunction-induced senescence (MiDAS). In these cells, MiDAS is mediated through a NAD/AMPK/p53 axis that does not involve oxidative stress or nuclear DNA damage ^23^. Furthermore, in a rodent model of mitochondrial dysfunction, POLG ^D257A^ mice harboring a mutation in the proofreading domain of mitochondrial DNA polymerase PolG accumulate senescent cells with a similar MiDAS phenotype ^23^. It is important to note that while all senescence stimuli induce irreversible cell cycle arrest, the effector pathways and resulting senescent cell phenotypes are highly context-dependent.

At the molecular level, senescence is regulated by a multitude of signaling pathways ^1^ that are activated in response to distinct senescence stimuli. However, induction and maintenance of senescence ultimately impinge on two critical tumor suppressor pathways governed by p16 ^INK4a^/pRb and p53/p21. Sustained activation or overexpression of these cell cycle regulators or their downstream effectors induces senescence in experimental model systems ^24, 25^. The near ubiquitous disruption of these pathways in tumors and the increased cancer susceptibility arising from germline mutations in p53 and p16 ^INK4a^ highlight the importance of senescence as a tumor suppression mechanism ^26– 28^.

While inhibition of proliferation early in senescence results from cell cycle checkpoint activation, the stable irreversibility of the senescent state appears to be due, in part, to changes in higher-order chromatin structure. Senescence-related chromatin structures are a feature of some, but not all, senescent cells, and the formation of each structure is likely to be influenced by cell type, species, and senescence stimulus. Senescence-associated heterochromatic foci (SAHFs) were first observed in senescent human fibroblasts *in vitro* and may function to lock proliferative genes into transcriptionally inactive constitutive heterochromatin ^29, 30^. SAHF formation requires a functional p16/Rb unit and is modulated by the histone chaperone proteins histone repressor A (HIRA) and anti-silencing factor 1a (ASF1a) ^31– 33^. More recently, senescence-associated distension of satellites (SADS) has been documented during both replicative senescence and OIS as well as in benign pancreatic intraepithelial neoplasia ^34^. Stable chromatin changes in response to DDR-induced senescence have also been identified in ‘DNA segments with chromatin alterations reinforcing senescence’ (DNA-SCARS) that contribute to the maintenance of cell cycle arrest and secretion of the inflammatory cytokine interleukin-6 (IL-6). These SCARS, which are conserved in human and mouse cells, may represent organizing centers of persistent DDR signaling that further reinforce the senescence response ^35^.

## The senescence-associated secretory phenotype and the double-edged sword of senescence

It is now appreciated that senescence is more multi-dimensional than simply permanent cell cycle arrest. During the establishment of the senescent state, cells undergo complex and dynamic changes in morphology, metabolism, chromatin organization, and transcription. In response to many, but not all, stimuli, senescent cells develop a senescence-associated secretory phenotype (SASP) ^36, 37^. Also known as the senescence-messaging secretome ^38^, the SASP is composed of more than 40 secreted factors, including mitogens, immunomodulatory chemokines and cytokines, extracellular matrix (ECM)-remodeling proteases (matrix metalloproteinases), and ECM/insoluble proteins (reviewed in 39). Not all SASP components are upregulated in every senescent cell, and the precise complement of SASP factors depends on both the cell type and nature of the senescence stimulus ^36, 39^. Upregulation of SASP gene expression is modulated by several factors, including nuclear factor kappa B (NF-κB), c/EBPβ, and GATA4 ^40– 45^. A mechanistic link between chromatin remodeling during senescence and induction of SASP genes was recently identified in human fibroblasts undergoing OIS in response to oncogenic HRAS ^46^. Chromatin immunoprecipitation-sequencing (ChIP-Seq) analysis of proliferating, quiescent, or senescent IMR90 fibroblasts using H3K27Ac as a marker of active enhancers identified a subset of super-enhancers activated during senescence that correlate with a SASP transcriptional profile ^46^. Enrichment of the transcriptional co-activator BRD4 was both observed at senescence-activated enhancers and required for induction of the SASP during OIS ^46^. As the complex nature of the SASP continues to be delineated, it is likely that additional mechanisms of crosstalk between effector pathways of senescence will be uncovered.

The ability of the SASP to exert paracrine effects on neighboring cells is central to the dual nature of senescence. Whether these effects are beneficial or detrimental is highly context-dependent, influenced by the nature of the senescence stimulus, cellular context, and duration and composition of the SASP response. Autocrine reinforcement of the senescent growth arrest is accomplished through the activities of multiple SASP components ^47– 49^ as well as through the activation of inflammatory networks and chemokine signaling ^45, 50^. The SASP can exert its tumor-suppressive effects in a non-cell-autonomous manner by attracting and activating immune cells to generate both an innate and adaptive anti-tumor immune response. Recruitment and activation of T cells and natural killer (NK) cells to the tumor microenvironment or altered polarization of macrophages results in elimination of senescent or damaged cells ^47, 51– 53^ in a process termed ‘senescence surveillance’. Senescence surveillance of tumor cells has been shown to restrain tumorigenesis in a mouse model of hepatocellular carcinoma. Secretion of cytokines by senescent pre-malignant hepatocytes driven to OIS by NRASG12V expression resulted in immune clearance of senescent cells, dependent upon antigen-specific CD4 ^+^ T cells ^48^. Furthermore, disabling immune surveillance of senescent hepatocytes in severe combined immunodeficient (SCID) mice was sufficient to promote development of liver cancer ^48^.

The pro-tumorigenic effects of senescence are believed to be largely due to deleterious influences of the SASP on pre-malignant cells as well as on the tumor microenvironment. Senescent stromal cells are a rich source of secreted growth factors that can stimulate the proliferation of nearby parenchymal cells. Senescent fibroblasts have been shown to promote growth of pre-malignant and malignant breast epithelial cells, prostate epithelial cells, keratinocytes, and melanocytes ^36, 54^. While the SASP is known to have beneficial roles in facilitating tissue repair through the activity of ECM-degrading proteases ^55^, remodeling of the ECM can relax the structure of the tumor microenvironment, potentially favoring tumor cell motility, invasion, and metastasis. In addition to enhancing the invasiveness of epithelial cell types through the secretion of chemokines and matrix-degrading proteases, senescent stromal cells can induce an epithelial-to-mesenchymal transition that is classically associated with neoplastic progression ^56, 57^. Induction of the SASP has also been shown to promote metastasis of breast cancer cells to the bone ^58^. Using the fibroblasts accelerate stromal-supported tumorigenesis (FASST) mouse model, in which tamoxifen-inducible Cre is used to conditionally drive p27 ^KIP1^-dependent senescence under the control of tissue-specific promoters, Luo *et al*. demonstrated that senescent osteoblasts created a favorable microenvironment for seeding of murine breast tumor cells, thus enhancing their metastasis to the bone ^58^. Tumor cell dissemination required the SASP factor IL-6, as intracardiac injection of IL-6-neutralizing antibodies resulted in a striking reduction in metastatic tumor burden ^58^.

As discussed above, secretion of inflammatory cytokines and immune modulatory SASP components by senescent cells is central to the immune surveillance branch of tumor suppression. However, cytokines released by senescent cells can also contribute to the generation of an immunosuppressive microenvironment that favors tumor outgrowth. Mice exhibiting conditional Pten loss in the prostatic compartment develop invasive adenocarcinoma despite the induction of a strong senescence response ^59^. Although the Pten loss-induced cellular senescence was accompanied by a SASP that included chemoattractant cytokines, the resulting tumors exhibited infiltration by CD11b ^+^Gr1 ^+^ myeloid-derived suppressor cells that inhibited proliferation of CD8 ^+^ cytotoxic T cells and activation of NK cells, thus creating an immunosuppressive, pro-tumorigenic microenvironment ^59^. Using the FASST mouse model to drive p27 ^KIP1^-dependent senescence in the stromal compartment of the skin, Ruhland *et al*. demonstrated that senescent stromal cells drive inflammation, consistent with the development of a pro-inflammatory/SASP gene expression profile, likely through enhanced infiltration of immunosuppressive myeloid cells and reduced cytolytic CD8 ^+^ T cell responsiveness ^60^. The resulting immunosuppressive microenvironment was shown to promote tumor cell growth, dependent upon the production of IL-6 by the senescent stroma ^60^.

It is important to note that the immunosuppressive effects of senescence are not limited to senescent tumor or stromal cells. Accumulating evidence indicates that immune cell senescence contributes to suppression of anti-tumor immunity, thus facilitating immune escape. Senescence of responder naïve/effector T lymphocytes can be induced *in vitro* by regulatory T cells (Tregs) as well as tumor cell lines and primary tumor cells ^61, 62^. At the molecular level, induction of T cell senescence by Tregs is governed by the p38 and ERK1/2 branches of the MAPK signaling pathway ^61^, while tumor cells can elicit a senescent phenotype in naïve/effector T cells through intercellular transfer of cAMP which can be inhibited via ILR8 signaling ^62^. Thus, while senescence is intrinsically growth-suppressive as a result of permanent mitotic exit, in many cases immune suppression, whether driven by the SASP, immune cells, or tumor cells, may prime the tumor microenvironment to promote the outgrowth of increasingly malignant cells.

## Therapy-induced senescence: friend or foe?

Senescence can suppress tumorigenesis not only by limiting the malignant transformation of pre-neoplastic cells but also by halting the proliferation of tumor cells. In addition to apoptosis, senescence of tumor (and stromal) cells is induced by many therapeutic approaches, including radiation, chemotherapeutics, and small-molecule inhibitors. Strategies for therapy-induced senescence (TIS) can be divided into three broad categories: (a) activation of DDR-related senescence by radiation or genotoxic chemotherapeutics, (b) re-activation of senescence-mediated tumor suppressor genes, and (c) cytokine-induced senescence (CIS).

DNA-damaging agents are well known to induce senescence in tumor cells; however, most standard genotoxic chemotherapeutic regimens have proven unsuccessful in curing patients of their disease. While increased mutational load in tumor cells surviving genotoxic chemotherapy may contribute to chemoresistance, accumulating evidence suggests that chemotherapy-induced DNA damage can have unintended tumor-promoting consequences related to DDR-induced senescence and stromal effects of chemotherapy. The SASP was first defined as essentially a DDR response ^36^; therefore, it is plausible that standard chemotherapies could induce a potentially deleterious SASP. Using the Eμ-myc model of Burkitt’s lymphoma, Gilbert and Hemann showed that doxorubicin induced a senescence response in the thymic stroma accompanied by an acute increase in thymic IL-6 levels in non-tumor-bearing mice and lymphoma-bearing mice ^63^. Moreover, doxorubicin-induced IL-6 secretion was found to be chemoprotective in hepatocellular carcinoma cells, and chemoprotection could be ameliorated by inhibition of Jak2/3 signaling downstream of IL-6 ^63^.

Numerous genetic mouse models have been used to achieve tumor regression by activating senescence mediators or de-activating oncogenes (reviewed in 64). Tumor cell senescence can be induced by re-activation or re-expression of p53, inactivation of Myc or Bcr-Abl, and Pten inhibition ^59, 65, 66^, all of which have been shown to potentiate growth inhibition or tumor regression. Given the frequency with which the p16 ^INK4a^/pRb and ARF/p53 functional units are disrupted in tumor cells, pharmacological inhibition of upstream negative regulators of pRb and p53 is an attractive therapeutic approach. The last several years have seen the advent of targeted cyclin-dependent kinase 4 and 6 (cdk4/6) inhibitors. Cdk4 and cdk6 function as negative regulators upstream of pRb to promote cell cycle progression, and loss of p16 ^INK4a^ function can lead to enhanced Cdk4/6 pathway activation ^67^. Three selective cdk4/6 inhibitors performed well in clinical trials: LEE011/ribociclib/Kisqali (Novartis), palbociclib (IBRANCE, Pfizer Inc.), and abemaciclib (Eli Lilly and Company). Palbociclib was granted accelerated US Food and Drug Administration (FDA) approval in combination with letrozole for the treatment of estrogen receptor-positive, HER2-negative breast cancer ^68^, followed by breakthrough approval of abemaciclib and most recently FDA approval of ribociclib/Kisqali for similar indications as combinatorial therapy. Palbociclib has been shown to induce tumor cell senescence in several pre-clinical models ^69– 73^. Although there is currently no clinical evidence that palbociclib-induced senescence could promote tumorigenesis, co-injection of fibroblasts induced to senesce by palbociclib has recently been shown to potentiate *in vivo* growth of mouse melanoma cells ^74^. Of note, the authors demonstrated that palbociclib-induced fibroblast senescence is accompanied by robust induction of the SASP that is mediated by NF-κB but does not involve the DDR ^74^.

In contrast to cell-autonomous mechanisms of TIS induced by genotoxic stress or re-activation of growth-suppressive pathways, CIS limits tumor cell proliferation through non-cell-autonomous pathways. In β-cancer cells lacking functional pRb and p53 due to expression of SV40 T-antigen under the control of the rat insulin promoter (RIP-Tag), T _H_1 cytokines interferon gamma (IFN-γ) and tumor necrosis factor (TNF) induce senescent growth arrest *in vitro*, accompanied by restoration of functional p16 ^INK4A^/pRb ^75^. Adoptive transfer of T antigen–specific T _H_1 cells also induced senescence of tumor cells *in vivo*, resulting in reduced tumor burden ^75^. Likewise, tumor-targeted IL-12 induced both cytolytic killing and senescent growth arrest of human rhabdomyosarcoma xenografts in lethally irradiated mice reconstituted with a humanized immune system ^76^. The identification of CIS as a tumor-suppressive mechanism indicates a novel, non-cytolytic role for the immune system in restraining tumor cell proliferation.

Unlike apoptotic cells that are readily cleared by the immune system, TIS cells that are not removed by the immune system may accumulate over time. TIS cells share many similarities with senescent cells induced by non-chemotherapeutic stimuli, and persistent senescent cells indeed may be a reservoir for pro-inflammatory and pro-tumorigenic SASP mediators ^36, 77^. The relationships between persistent TIS cells and response to chemotherapy and disease outcome were examined in a recent study using p16-3MR mice, in which p16 ^INK4A^-positive senescent cells can be selectively ablated using ganciclovir ^77, 78^. Chemotherapeutic agents with varying mechanisms of action were all shown to be capable of inducing TIS *in vivo*, and removal of senescent cells with ganciclovir five days after administration of doxorubicin ameliorated the expression of pro-inflammatory SASP components induced by chemotherapy. Importantly, removal of senescent cells in the p16-3MR mouse model limited not only chemotherapy-induced fatigue but also tumor relapse and metastasis. Additionally, the prevalence of p16 ^INK4A^-positive senescent cells in peripheral blood T cells obtained from women prior to undergoing chemotherapy for breast cancer successfully predicted risk of chemotherapy-related fatigue ^78^. This elegant study provides further impetus for the development of senolytic compounds to selectively target senescent cells or the SASP itself ^78, 79^.

Potential strategies for ameliorating the deleterious effects of senescence have been proposed ^80– 83^ with the overall goal of maintaining the anti-proliferative effects of senescence while harnessing the pro-tumorigenic activities of the SASP. Broadly speaking, these approaches include selectively targeting and eliminating senescent cells, preventing the development of the SASP, or targeting the SASP in existing senescent cells (reviewed in 80). The recently developed senolytic compound ABT-263 (navitoclax) is a potent inhibitor of BCL2 and BCL-XL that induces caspase-dependent apoptosis of senescent cells. Selective removal of senescent cells by navitoclax has been shown to rejuvenate the hematopoietic stem cells and muscle stem cell compartments in aged or lethally irradiated mice ^79^; however, the clinical use of this potent senolytic thus far has been limited by its thrombocytopenic effects ^84^. Modulation of the SASP has yielded promising results in Pten-null mouse prostate tumors, in which prostate-specific genetic ablation of Stat3 reduced expression of tumor-suppressive chemokines while sparing expression of chemoattractants ^59^. Although combined Pten and Stat3 deficiency did not prevent tumorigenesis, tumors that did develop were smaller and less invasive, with increased infiltration of immunostimulatory CD8 ^+^ T cells, NK cells, and B cells ^59^. The work of Toso *et al*. provides *in vivo* proof of principle that the pro-tumorigenic effects of senescence can be manipulated toward a tumor-suppressive phenotype ^59^.

## Conclusions

Given that senescence can act as a double-edged sword in tumorigenesis, it is imperative to consider its dual nature in the design and use of senescence-inducing anti-cancer therapies. Future studies aimed at uncoupling the detrimental effects of senescence from its anti-proliferative effects may permit fine-tuning of the senescence response to favor good clinical outcomes. A deeper understanding of the molecular and immunological contexts that determine a tumor-suppressive versus a tumor-promoting SASP will aid in the development of senescence-targeting therapies. Combination therapies consisting of a TIS arm to halt tumor cell proliferation and a senolytic arm to rein in the SASP through removal of senescent cells may ultimately be of great clinical benefit.
